# Duffy blood system and G6PD genetic variants in vivax malaria patients from Manaus, Amazonas, Brazil

**DOI:** 10.1186/s12936-022-04165-y

**Published:** 2022-05-08

**Authors:** Natália S. Ferreira, Jéssica Lorena S. Mathias, Sérgio Roberto L. Albuquerque, Anne Cristine G. Almeida, Ana C. Dantas, Fernanda C. Anselmo, Emerson S. Lima, Marcus Vinicius G. Lacerda, Paulo A. Nogueira, Rajendranath Ramasawmy, Marilda S. Gonçalves, José P. Moura Neto

**Affiliations:** 1 Universidade Do Estado Do Amazonas—Fundação Hospitalar de Hematologia E Hemoterapia Do Amazonas, Manaus, Amazonas Brazil; 2 Universidade Federal Do Amazonas, Faculdade de Ciências Farmacêuticas, Avenida General Rodrigo Otávio Jordão Ramos, Manaus, Amazonas 6200 Brazil; 3 Fundação de Medicina Tropical Dr. Heitor Vieira Dourado, Manaus, Amazonas Brazil; 4 Instituto Leônidas E Maria Deane, FIOCRUZ, Manaus, Amazonas Brazil; 5 Faculdade de Medicina, Universidade Nilton Lins, Manaus, Amazonas Brazil; 6 Instituto Gonçalo Moniz, Salvador, Bahia Brazil

**Keywords:** Severe malaria, G6PD, Duffy system, *P. vivax*

## Abstract

**Background:**

Over a third of the world’s population is at risk of *Plasmodium vivax*-induced malaria. The unique aspect of the parasite’s biology and interactions with the human host make it harder to control and eliminate the disease. Glucose-6-phosphate dehydrogenase (G6PD) deficiency and Duffy-negative blood groups are two red blood cell (RBC) variations that can confer protection against malaria.

**Methods:**

Molecular genotyping of G6PD and Duffy variants was performed in 225 unrelated patients (97 with uncomplicated and 128 with severe vivax malaria) recruited at a Reference Centre for Infectious Diseases in Manaus. G6PD and Duffy variants characterizations were performed using Real Time PCR (qPCR) and PCR–RFLP, respectively.

**Results:**

The Duffy blood group system showed a phenotypic distribution Fy(a + b−) of 70 (31.1%), Fy(a + b +) 96 (42.7%), Fy(a−b +) 56 (24.9%) and Fy(a−b−) 1 (0.44%.) The genotype FY*A/FY*B was predominant in both uncomplicated (45.3%) and severe malaria (39.2%). Only one Duffy phenotype Fy(a-b) was found and this involved uncomplicated vivax malaria. The G6PD c.202G > A variant was found in 11 (4.88%) females and 18 (8.0%) males, while c.376A > G was found in 20 females (8.88%) and 23 (10.22%) male patients. When combined GATA mutated and c.202G > A and c.376A > G mutated, was observed at a lower frequency in uncomplicated (3.7%) in comparison to severe malaria (37.9%). The phenotype Fy(a−b +) (p = 0.022) with FY*B/FY*B (p = 0.015) genotype correlated with higher parasitaemia.

**Conclusions:**

A high prevalence of G6PD c202G > A and c.376A > G and Duffy variants is observed in Manaus, an endemic area for vivax malaria. In addition, this study reports for the first time the Duffy null phenotype Fy(a-b-) in the population of the Amazonas state. Moreover, it is understood that the relationship between G6PD and Duffy variants can modify clinical symptoms in malaria caused by *P*. *vivax* and this deserves to be further investigated and explored among this population.

**Supplementary Information:**

The online version contains supplementary material available at 10.1186/s12936-022-04165-y.

## Background

Malaria is one of the most serious public health problems worldwide, and in the tropical and subtropical regions, this parasitic disease is the leading cause of social and economic problems [1, 2]. In 2020, 140,974 cases of malaria were recorded in Brazil, with a total number of *Plasmodium vivax* cases of 118,651 (84.2% of the total cases) and *Plasmodium falciparum* of 22,182 (15.8%) [3].

Susceptibility or resistance to malarial infection depends on the host-parasite interactions, the *Plasmodium* sp., the parasite load, the host genetics factor, the immunological status of the host [4, 5]. The clinical manifestation of malaria may include severe anaemia, coagulation disorders, prominent thrombocytopenia, and numerical or functional alterations in leukocytes with spleen involvement [6]. In regions where *P. vivax* infections are endemic, clinical complications and mortality are reported and this has led to the characterization of *P. vivax* malaria as a serious or even fatal disease [7, 8].

Although little is known of the pathophysiology, the progression and aggravation of *P. vivax* malaria is mainly associated with anaemia, which is occasionally due to severe haemolysis [9–11]. Other pathophysiological events, such as oxidative stress, may influence the development of clinical conditions [12, 13].

The Duffy Antigen Receptor for Chemokines (DARC), recently renamed Atypical Chemokine Receptor 1 (ACKR1), acts as a possible facilitator in the process of erythrocyte invasion by *P. vivax*, and blood system antigens act as receptors for *P. vivax* merozoite ligands that contain Duffy-Binding-Like (DBL) domains through alleles. These Duffy blood group system antigens (Fy^a^, Fy^b^, Fy3, Fy5, and Fy6) are encoded by two co-dominant allelic forms FY*A and FY*B that differ by the single nucleotide polymorphism (SNP) in position c.125G > A of the exon 2 [14]. The SNP located at position c.-67 T > C in the GATA promoter region is characterized by the allele FY*BN.01 that silences Fy^b^ expression in erythroid cells, and homozygous individuals (FY*BN.01 / FY*BN.01) bear the Fy(a−b−) phenotype that is known to be a protective factor against vivax malaria infections [15].

Another phenotype associated with weak Fy^b^ antigen expression is determined by SNP c.265C > T and c.298G > A. These polymorphisms occur within the first intracellular loop of the Duffy protein, which result in reduced expression of the Fy^b^ antigen. The frequency of Fy^b weak^_,_ characterized as FY*BW.01 is approximately 2% in Caucasians [16]. Some polymorphisms resulting in the Fy^a weak^ (FY*AW.01) allele are yet to be observed in inhabitants of the Amazonas state, Brazil [17, 18].

Glucose 6-phosphate dehydrogenase (G6PD) is an enzyme involved in the pentose monophosphate pathway. Deficiency of this enzyme leads to free radical-mediated oxidative damage to erythrocytes, thus causing haemolysis. G6PD deficiency, linked to the inheritance of X chromosome(s) with disease causing variant(s), is mostly prevalent in people of African, Asian, and Mediterranean descent [19, 20]. In females, there are selective advantages with G6PD A-, which is characterized by the combination of variants A376G (c.376A > G) with variants G202A (c.202G > A), A542T, G680T or T968C [21, 22]. It is suggested that the heterozygous state offers women a selective advantage against severe malaria [23–25], and it is known that G6PD deficiency has a prevalence of 8% in malaria-endemic regions [26].

The clinical severity between malaria endemic areas can be complex with multifactorial influence and genetic factors [27]. In endemic areas of *P. vivax* malaria, comorbidities such as inherited haemoglobinopathies and G6PD deficiency require investigation since they may also be factors that affect heterogeneity in clinical manifestations, and may be characterized by subtypes or endophenotypes [28].

Vivax malaria is a challenge for malaria control strategies and its elimination [29]. The unique parasite biology that involves the formation and subsequent reactivation of latent forms in the liver, and the ability of *P. vivax* to infect the vector before symptoms occur, favours the perpetuation of the parasitic life cycle and, due to subpatent infections, this causes difficulty in tracking infected individuals [30]. Many studies show the G6PD c.202G > A and c.376A > G variants and Duffy-negative blood group are two RBC variants that confer protection against malaria [19, 31, 32]. Many authors postulate that this is a result of the increased sensitivity of *P*. *vivax* to oxidative stress in G6PD deficiency, with negative influence on the parasite, as well as the association of different phenotypes of the Duffy blood group in the invasion of RBCs [33–35]. Furthermore, the mechanism of action between G6PD and Duffy group is not fully known. Based on these facts, this study aimed to determine the prevalence of Duffy alleles and G6PD c.202G > A and c.376A > G variants in uncomplicated and severe malaria patients, in order to answer if these erythrocyte variants deserve to be better investigated with clinical signs, susceptibility, protection and parasitaemia against of P. vivax infections.

## Methods

This is a retrospective study that is based on a convenience sample for the period between March 2013 and April 2016, which was obtained from the Tropical Medicine Foundation Dr. Heitor Vieira Dourado (FMT-HDV), a reference center for infectious diseases in Manaus, capital of the Amazonas state, Brazil. Blood samples were collected from all the participants. Inclusion criteria were patients over 18 years of age, of either sex and of any skin colour with severe (hospitalized) or uncomplicated (outpatient) malaria, and without any associated diseases. All patients were treated at the clinical research ward at the hospital. All the patients included in the study are unrelated individuals, non-smokers and non-diabetic. Patients with comorbidities, haemoglobinopathies, mixed *Plasmodium* infections, viral infections and pregnant women were excluded. Patients were classified as either uncomplicated malaria or severe malaria following the World Health Organization (WHO) recommendations (Additional file 1: Table S1) [36, 37].

Approximately 2.5 mL of peripheral blood were collected in tube with EDTA (ethylenediaminetetraacetic acid disodium salt) at a concentration of 1.5 mg/mL for blood counts. Aliquots of 0.3 mL blood in 1.5 mL tubes were kept for extraction of nuclear DNA. An additional 2.5 mL were collected in a tube without anticoagulants for biochemical analyses. Immediately after blood collection, haematological determinations were performed using an automated counter (ABX Pentra 80, Horiba Diagnostics, Montpellier, France) and biochemistry was performed on a Beckman Coulter (Inc, CA, USA).

The diagnosis of the malaria infection followed the standardized protocol established by FMT-HVD, which is a nationally recognized centre for the diagnosis of malaria. In summary, the malaria diagnosis was performed under light microscopy with multiple readings for the parasitaemia. Identification of *Plasmodium* species (*Plasmodium vivax, Plasmodium falciparum* and *Plasmodium ovale*) was done by polymerase chain reaction (PCR) and Real Time PCR (qPCR) methods [38]. The determination of parasitaemia was based on the count of asexual parasites per 200 or maximum 500 white blood cells. The total number of leukocytes of each patient was used for the determination of the parasite density using the following formula [18]:

$$ {\text{Parasite density/}}\mu {\text{L }} = \frac{{{\text{Number of parasites }} \times {\text{ Total white blood cell }}\left( {{\text{WBC}}} \right){\text{ counts}}}}{{{\text{Number of white blood cells }}\left( {{\text{WBC}}} \right){\text{ counted}}}} $$

### DNA analysis

DNA was extracted from 200 µL of whole blood according to the QIAamp DNA Mini Kit (Qiagen, Hilden, DE) manufacturer's protocol (Cat No./ID 51,304). After extraction, the DNA was quantified using a spectrophotometer (NanoDrop™ 2000, Thermo Fisher Scientific, Massachusetts, USA), and then stored at − 20 ºC.

### Duffy genotyping by PCR–RFLP

Duffy genotyping was performed using conventional PCR for amplification of the sequence of interest, followed by restriction enzyme digestion; one for Duffy blood group (Duffy PCR) and one for the GATA box variant (GATA PCR). The Fy(a − b −) phenotype is known as “erythrocyte silent” and arises from homozygosity FY*BN.01 allele from c.1-67 T > C mutation [15]. This mutation impairs promoter activity in erythroid cells by disrupting a binding site for the GATA-1 erythroid transcription factor, stopping Fyb antigen expression in red blood cells [39, 40].

Genotyping for Duffy blood system groups was performed with synthetic oligonucleotides FYAB1 (5 'TCC CCC TCA ACT GAG AAC TC 3') and FAB2 (5 'AAG GCT GAG CCA TAC CAG AC 3'). Amplified products were visualized on a 1.5% agarose gel stained with ethidium bromide. After PCR amplification was confirmed, the products were processed using *BanI* restriction enzyme digestion and incubated for at least 4 h at 37 ºC. The enzyme digestion product was electrophoresed on a 1.5% agarose gel stained with ethidium bromide for discrimination of the alleles.

Duffy genotypes, as well as phenotype characterizations, were named according to the FY (ISBT 008) Blood Group Alleles (http://www.isbtweb.org/fileadmin/userupload/Workingparties/WPonRedCellImmunogeneticsand/008FYAllelesv4.1.pdf) [41].

For verification of c.-67 T > C SNP in the GATA box promoter region, FY*A/FY*B and FY*B/FY*B genotype samples were used. Synthetic oligonucleotides FYN1 (5 'CAA GGC TGA CCC TA 3') and FYN2 (5 'CAT GGC ACC GTT TGG TTC AG 3') were used for the GATA PCR.

The GATA PCR product was treated with an *StyI* restriction enzyme and incubated for at least 4 h at 37 °C. The enzyme digestion product was observed on a 2.5% agarose gel. GATA normal genotypes appeared as 108 and 81 bp bands, while GATA mutated showed an additional 61 bp band. Samples with genotypes FY*A/FY*A and FY*A/FY*B were used to verify the SNPs c.265C > T and c.298G > A in the FY*AW.02 coding region and for Fy^x^.

The PCR product was treated with the restriction enzyme *MspAI* in order to verify the SNP c.265C > T, and was then incubated for 4 h at 60 °C. For the SNP c.298G > A, the *Mwol* restriction enzyme was used and then incubated for 4 h at 37 °C. Both were discriminated on an 8% polyacrylamide gel. The mutated genotypes for the SNP c.265C > T had an additional band of 161 bp, and c.298G > A had an additional band of 343 bp.

### G6PD genotyping

For characterization of the variants, Real Time PCR (qPCR) was performed (QuantStudio™ 3 Real-Time PCR System, Applied Biosystems, Thermo Fisher Scientific®) using TaqMan® probes that were specific for each polymorphism. The amplification reaction was performed for a final volume of 12 uL/reaction, which contained 5 μL of 2 × TaqMan Universal Master Mix, 0.3 μL of 20 × SNP genotyping assay, 4.8 μL of sterile water and 2.0 μL of DNA (~ 100 ng) of the sample. The G6PD variants analysed in this project were chosen based on globally observed frequencies and their clinical importance according to WHO classification. The inclusion of the G6PD variants c.202G > A and c.376A > G was based on several studies that showed that both account for more than 95% of all mutations demonstrated in the Brazilian population, as well as in the population of the Amazonas state. For the qPCR technique, the (A-) V68M (202G > A) (rs1050828) and (A +) N126D (376A > G) (rs1050829) probes were used. To confirm the mutations found, amplification by PCR of the relevant DNA segments was performed and was followed by DNA sequencing (ABI 3100, Applied Biosystems, Foster City, CA, USA) [42–44].

### Statistical analysis

Data were entered into a database using Graphpad-Prism 5.0 software (Graphpad Software, San Diego, CA, USA) and IBM SPSS Statistics, version 19 (IBM Corp., Armonk, NY, USA), and organized by variable type. The analysis of qualitative or categorical variables of three or more groups was performed using the non-parametric Chi-square test (χ2) test using Yates' continuity correction. For the analysis categorical or dichotomous data was used Fisher's exact test when the number of subjects per cell was less than 5. All the statistical tests were performed using a significance level of 5%. The Hardy–Weinberg equilibrium (HWE) was evaluated by comparing expected genotypic frequencies with the observed ones using a Chi-square test.

## Results

### Clinical and laboratory data

A total of 225 patients diagnosed with *P. vivax* malaria were included in the study. Ninety-seven patients (43.1%) had uncomplicated malaria and 128 (56,9%) had severe malaria. Table 1 showed the epidemiological and physical parameters for uncomplicated and severe *P. vivax* malaria stratified by gender. Most of the severe malaria male cases had pallor (82.5%), choluria (77.8), headache (76.2%), loss of appetite (74.6%) and hepatosplenomegaly (71.4%). Among the female patients, headache (92.9%), loss of appetite (85.7%), pallor (83.3%), vomiting (83.3%) and choluria (73.8%) were the common symptoms.

**Table 1:** Epidemiological clinical parameters for uncomplicated and severe *P. vivax m*alaria patients from Manaus, Amazonas state

Clinical Signs	Female			Male		
	Severe (n = 60)	Uncomplicated (n = 47)	p-value	Severe (n = 68)	Uncomplicated (n = 50)	p-value
Abdomen pain	43	35	NS	44	33	NS
Severe anaemia	13	1	.002 ^*^	11	1	< .001 ^*^
Loss of appetite	51	35	NS	5	37	NS
Choluria	44	23	.008 ^**^	53	21	< .001 ^*^
Cough	24	16	NS	21	16	NS
Diarrhea	17	12	NS	28	18	NS
Dyspnea	20	23	NS	24	18	NS
Epistaxis	3	5	NS	3	10	.008 ^*^
Headache	56	39	NS	51	46	.013 ^**^
Hemoglobinuria	11	6	NS	14	3	.021 ^*^
Hepatosplenomegaly	39	20	.016 ^*^	49	14	< .001 ^**^
Jaundice	43	9	< .001 ^**^	43	12	< .001 ^**^
Leukocyturia	20	18	NS	30	8	< .001 ^**^
Obesity	5	5	NS	9	1	.028 ^*^
Oliguria	09	4	NS	10	4	NS
Pallor	50	33	NS	56	43	NS
Petechia	3	11	.005 ^*^	3	5	NS
Vomiting	50	40	NS	44	36	NS
Whole blood transfusion	10	4	NS	18	3	< .001 ^*^

It show little difference in clinical signs between genders when diagnosed with uncomplicated and severe vivax malaria. However, male patients most frequently report symptoms when they have severe malaria

N: Cases. Results are based on ^*^ χ2 test (Yates’s corrections) and ^**^ Fisher Exact Test. NS: no statistically significant association (P > 0.05). Statistically significant associations (P < 0.05) are emphasized in bold type

Patients with severe malaria presented a significant decrease in RBCs (female 3.51 and 3.48 male × 10^6^ uL), haemoglobin (female 9.72 and male 10.06 g/dL) and haematocrit (female 30.45 and male 30.22%). High bilirubin levels were also observed (12.5, 10.36, female 12.5 and male 10.36) (ANOVA—p < 0.001). Hypoglycaemia (< 70 mg/dL), a clinical and laboratory features of severe malaria, was detected in 16% of patients with severe malaria (Table 2).

**Table 2:** Hematological and biochemical parameters for uncomplicated and severe *P. vivax* malaria patients from Manaus, Amazonas state

	FEMALE			MALE		
	Severe (n = 60)	Uncomplicated (n = 47)	p-value	Severe (n = 68)	Uncomplicated (n = 50)	p-value
Age, median	37.07 ± 12.45	35.36 ± 13.76	NS	39.43 ± 13.45	25.01 ± 19.39	NS
Temperature, median	37.25 ± 0.79	37.13 ± 0.89	NS	36.95 ± .74	36.62 ± 0,91	NS
Weight (Kg)	56.47 ± 17.91	62.45 ± 16.09	NS	51.89 ± 24.07	58.30 ± 15.90	NS
Numbers of days in hospital	3.34 ± 3.19	3.09 ± 1.78	NS	5.25 ± 6.97	3.01 ± 2.24	NS
Asexual Parasite Density	16,482.6 ± 21,702.3	7,570.21 ± 8,381.54	**.018**	16,274.01 ± 23,747.9	5,063.79 ± 9,019.13	**.031**
Parasitaemia	344.04 ± 194.48	294.93 ± 201.68	NS	259.98 ± 190.92	247.16 ± 204.44	NS
Platelet Count × 10^9^/L	59.94 ± 46.06	87.36 ± 77.78	NS	120.08 ± 107.65	124.51 ± 103.95	NS
WBC × 10^3^ uL	5,187.5 ± 1,971.7	5,975.5 ± 2,451.6	NS	8,026.5 ± 4,626.9	5,830.7 ± 2,354.1	**.011**
RBC × 10^6^ uL	3.53 ± 0.89	3.98 ± 0.71	**.011**	3.37 ± 1.18	4.33 ± 0.78	** < .001**
Haemoglobin (g/dL)	10.08 ± 2.74	11.01 ± 2.15	NS	9.66 ± 3.01	12.19 ± 2.32	** < .001**
Haematocrit (%)	30.76 ± 7.8	33.95 ± 6.67	**.046**	29.06 ± 9.11	37.84 ± 7.32	** < .001**
MCV (fL)	87.43 ± 5.77	85.08 ± 6.35	NS	87.48 ± 9.78	87.29 ± 6.37	NS
MCH (pg)	28.84 ± 2.94	27.56 ± 2.13	**.023**	29.16 ± 3.59	28.16 ± 2.27	NS
MCHC (g/dL)	32.94 ± 1.76	32.43 ± 1.49	NS	33.32 ± 1.63	32.28 ± 1.68	**.002**
Reticulocytes (%)	2.04 ± 2.21	1.64 ± 1.51	NS	4.46 ± 4.69	3.22 ± 3.88	NS
Direct bilirubin (mg/dL)	4.71 ± 2.71	0.53 ± 0.56	** < .001**	3.11 ± 4.46	0.86 ± 2.19	**.024**
Indirect bilirubin (mg/dL)	2.85 ± 4.39	.081 ± 0.61	**.008**	4.65 ± 6.88	1.14 ± 2.15	**.018**
Total bilirubin (mg/dL)	6.32 ± 4.41	1.34 ± 0.78	** < .001**	7.98 ± 10.05	1.12 ± 0.68	** < .001**
Urea (mg/dL)	33.06 ± 14.20	30.63 ± 16.84	NS	61.45 ± 35.63	36.86 ± 13.75	** < .001**
Creatinine (mg/dL)	0.81 ± 0.34	0.88 ± 0.38	NS	1.40 ± 0.94	1.02 ± 0.34	**.022**
Lactate dehydrogenase (U/L)	855.86 ± 339.73	548.95 ± 173.95	** < .001**	921.06 ± 524.01	650.07 ± 235.41	NS
Alkaline Phosphatase (IU/L)	260.41 ± 169.77	251.24 ± 148.09	NS	232.53 ± 132.78	127.85 ± 114.66	** < .001**
Ca ^++^ (mM)	8.64 ± 0.80	8.85 ± 0.99	NS	8.73 ± 1.21	9.33 ± 0.89	NS
K ^+^ (mM)	3.66 ± 0.49	3.92 ± 0.41	NS	3.99 ± 0.59	3.97 ± 0.45	NS
Glucose mg/dL	88.82 ± 32.71	97.58 ± 28.06	NS	99.20 ± 35.03	101.44 ± 33.65	NS

It shows few significant values in haematological, biochemical and physical parameters between genders when diagnosed with uncomplicated and severe vivax malaria. However, hematological values were the lowest in male patients when they have severe malaria

Results are based on ANOVA and Bonferroni corrected post-hoc t-test. Continuous variables are presented as mean ± SD

*N* Cases, *WBC* White Blood Cells, *RBC* Red Blood Cells, *MCV* Mean corpuscular volume, *MCH* Mean corpuscular haemoglobin, *MCHC* Mean corpuscular haemoglobin concentration, NS: no statistically significant association (P > 0.05). Statistically significant associations (P < 0.05) are emphasized in bold type

### Duffy genotyping

**Fig. 1:**
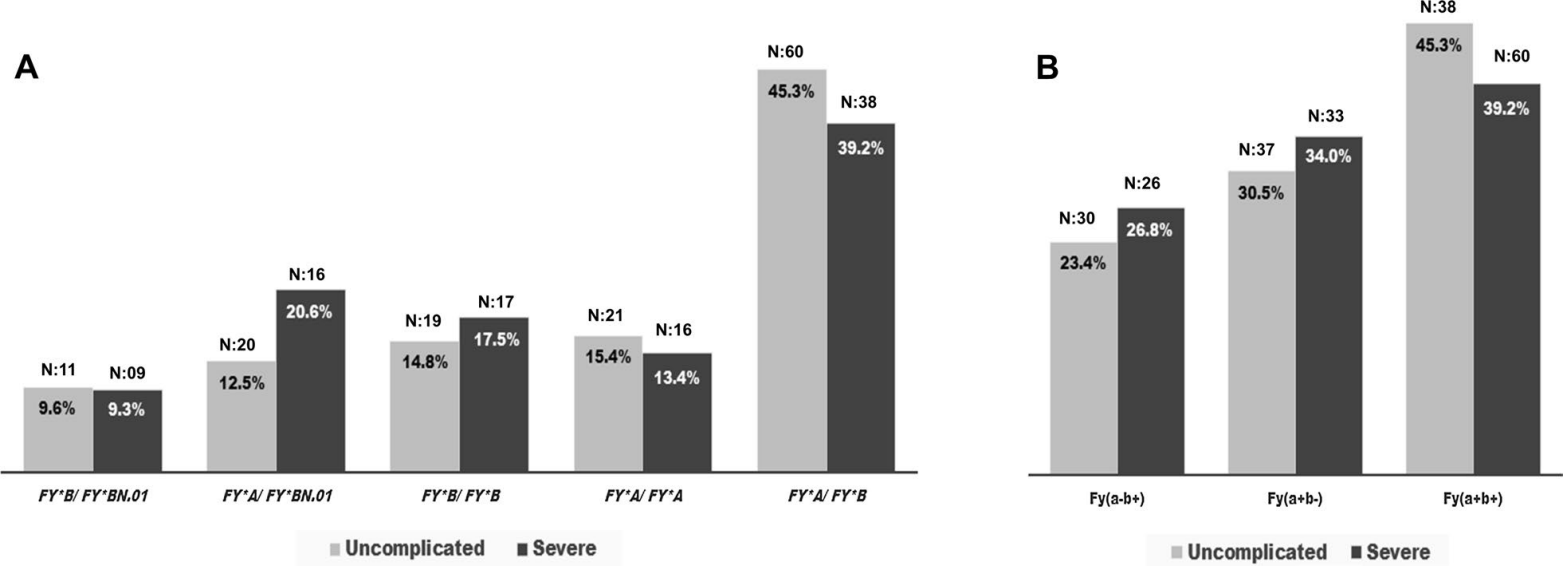
Genotypic (**A**) and phenotypic (**B**) distribution of Duffy antigens among uncomplicated and severe *P. vivax* malaria patients. Highest frequency for genotypes (FY*A/FY*B) and phenotypes Fy(a−b +) with null and weak expression of Duffy system were found in both uncomplicated and severe malaria patients

Analysis of the Duffy system (Table 3) showed a phenotypic distribution of Fy(a + b-) of 31.1% (70/225), Fy(a + b +) 42.70% (96/225), Fy(a-b +) 24. 89% (56/225) and Fy(a-b-) 0.44% (1/225). The genotype FY*A/FY*B was predominant in both uncomplicated (45.3%) and severe malaria (39.2%) (Fig. 1). The phenotypic frequency distribution of Duffy genotypes among uncomplicated and severe vivax malaria by gender is shown in Additional file 2: Table S2.

**Table 3:** Allele frequency of the Duffy blood group from *P. vivax* malaria patients

Phenotypes	Genotypes	Percentage N (%)
Fy(a + b +)	FY*A / FY*B	96 (42.70)
Fy(a + b−)	FY*A / FY*BN.01	36 (16.0)
	FY*A / FY*A	34 (15.10)
Fy(a−b +)	FY*B / FY*B	36 (16.0)
	FY*B / FY*BN.01	20 (8.88)
Fy(a + ^w^)	FY*A / FY*AW.02	02 (0.88)
Fy(a−b−)	FY*BN.01 / FY*BN.01	1 (0.44)

The phenotypes, respective alleles and their distribution percentage among *vivax* malaria patients is shown. The FY*A/FY*B genotype was more frequent than the null expression genotype in the vivax malaria patients

*N* Cases

### G6PD genotyping

Twenty-nine patients were carriers of the G6PD c.202G > A variant; 18 (8.0%) males were hemizygous, 10 (4.44%) and 1 (0.44%) female was heterozygous and homozygous, respectively. The c.376A > G was identified in 43 (19.11%) patients. Eighteen women (8.0%) were heterozygous and 2 (0.88%) were homozygous. Twenty-three (10.22%) males were hemizygous. Both SNPs were in HWE as calculated from the frequencies of the alleles among the females, since the G6PD gene is located on the X chromosome.

### Duffy and G6PD genotypes with analysis of clinical characteristics

The FY*BN.01/FY*BN.01, responsible for the Fy(a−b−) phenotype, was observed in one patient (female) with uncomplicated malaria who was not a carrier of the G6PD variants. Among patients with uncomplicated malaria, the frequency of c.202G > A and c.376A > G variants was low, particularly when these polymorphisms were concomitant. Severe malaria patients showed no variations in frequency for the c.202G > A variant. However, in the presence of A376G, there was a slight decrease in frequency.

**Fig. 2:**
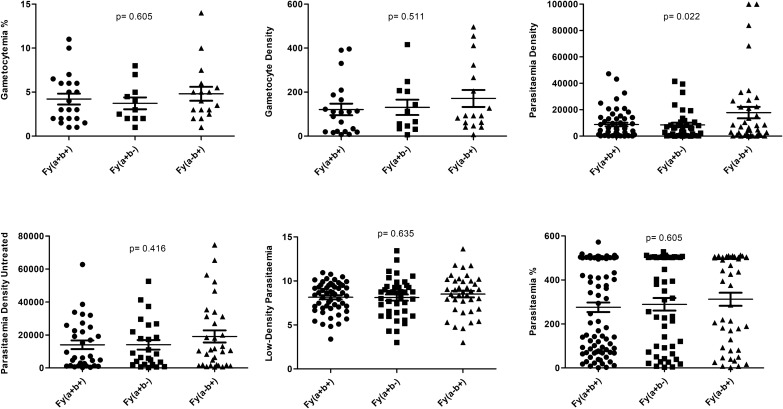
Parasitaemia in *P. vivax* malaria patients and Duffy phenotypes. There was statistical significance (p = 0.022) for parasitic density, mainly in the phenotype Fy(a−b +)

**Fig. 3:**
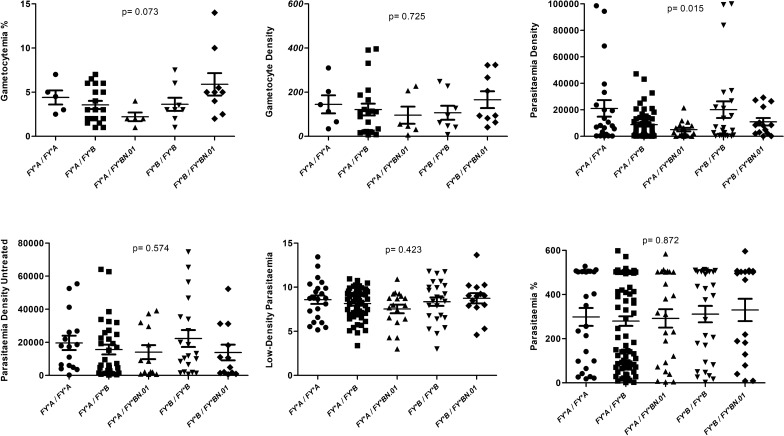
Parasitaemia in *P. vivax* malaria patients and Duffy genotypes. The FY*B/ FY*B genotype had the highest parasitic values and, when an FY*B allele was present and expressed, these values were higher. The parasitic density was lower in the presence of the GATA variant

When combined with the GATA variant, the c.202G > A and c.376A > G variants were observed at a lower frequency in uncomplicated (3.7%) malaria in comparison to severe malaria (37.9%) (Table 4). The levels of gametocytes and mean parasitaemia (± standard deviation), considering the number of parasites found per mm^3^, is shown in Figs. 2 and 3. The minimum and the maximum parasitaemia were 18/µL–101,904/µL among uncomplicated malaria and 29/µL–102,358/µL in severe malaria. Statistical significance (p = 0.022) was observed for parasitaemia. Higher parasitaemia was observed to phenotype Fy(a−b +) (p = 0.022) and genotypes FY*A/ FY*A and FY*B/ FY*B, with lower parasitaemia in the presence of the FY*A/FY*BN.01 (p = 0.015).

**Table 4:** Genotypic frequency distribution of G6PD variants among uncomplicated and severe P. vivax malaria patients by the presence of Duffy GATA normal or mutated variants

Malaria	Duffy GATA	G202A N (%)		p-value	A376G N (%)		p-value	G202A / A376G N (%)		p-value
		202wt	c.202^GA/AA^		376wt	c.376^AG/GG^		202wt/376wt	c.202^GA/AA^/c.376^AG/GG^	
Uncomplicated	Normal	95 (95.0)	5 (5.0)	.654 ^**^	89 (88.7)	11 (11.3)	.814 ^**^	89 (97.8)	2 (2.2)	.545 ^**^
	Mutated	25 (92.6)	2 (7.4)		25 (92.6)	2 (7.4)		26 (96.3)	1 (3.7)	
Severe	Normal	55 (80.1)	13 (19.1)	.309 ^*^	49 (72.1)	19 (27.9)	.463 ^*^	59 (86.8)	9 (13.2)	.013 ^*^
	Mutated	20 (69.0)	9 (31.0)		18 (62.1)	11 (37.9)		18 (62.1)	11 (37.9)	
Total	Normal	150 (89.3)	18 (10.7)	.135 ^*^	138 (82.1)	30 (17.9)	.493 ^*^	157 (93.5)	11 (6.5)	.058 ^*^
	Mutated	45 (80.4)	11 (19.6)		43 (76.8)	13 (23.2)		47 (83.9.0)	9 (16.10)	

The presence of the GATA (FY*BN.01) variant was associated with the c.202G > A and c.376A > G variants in patients with severe and uncomplicated vivax malaria. The presence of both the GATA and G6PD variants presented more frequently in cases of severe malaria; however, this finding did not present statistical significance, though there was a possible association with disease severity (p = 0.043)

*N* cases, *wt* Wild Type

^*^χ2 test (Yates’s corrections)

^**^Fisher’s exact test

## Discussion

Death due to malaria, regardless of the *Plasmodium* species, is not related to gender, though children and pregnant women are more susceptible to malaria. However, socio-economic and cultural factors play a central factor in determining differences in gender vulnerability to malaria [45–47].

Six (19.35%) female patients and sixteen (39.02%) male patients had both G6PD c.202G > A/c.376A > G mutations concomitantly c.202G > A and c.376A > G are responsible for the majority of the observed prevalence of G6PD deficiency in Brazil. The occurrence of these concomitant mutations, which can be in heterozygous or homozygous states in females and hemizygous in males, deserves attention in genetic association studies in order to investigate possible clinical phenotypes of this deficiency among patients receiving treatment with malaria medication [48–50].

The higher the parasitaemia in the host, the higher the consumption of glucose that may lead to hypoglycaemia, mainly in severe malaria. Hypoglycaemia may provoke dizziness, palpitations, tremors and loss of consciousness [51]. Careful glucose monitoring should be targeted in these complications, especially in those patients with G6PD deficiency. However, none of the G6PD alleles correlated with low levels of glucose among the severe malaria patients [52].

In this study, FY*A/FY*B was the most frequent genotype in both malaria patients, uncomplicated (45.3%) and severe (39.2%). These findings corroborate the study reported by Cavasini et al. [53] which correlated the high frequency of the FY*A and FY*B alleles among *P. vivax* malaria patients. The FY*A/FY*B and FY*A/FY*A genotypes were associated with a high frequency of *P. vivax* infection, thus suggesting that these individuals have a higher risk of developing malaria disease. The FY*A/FY*A and FY*A/FY*B genotypes are associated with increased frequency of *P. vivax* infection, while FY*A/FY*BW.01 and FY*B/FY*BW.01 were associated with low parasitic density levels [18].

The presence of a single case with null Duffy phenotype Fy(a−b−) is first described in the population of the Amazonas state. In endemic areas, the Duffy negative blood group is reported as a protective factor against *P. vivax* malaria infection [54]. In a study conducted in São Paulo, the phenotypic frequencies found in blood donors for Duffy blood system antigens were 19.8% for the phenotype Fy(a + b−) in Caucasians and 14.0% in African-Brazilians, Fy(a + b +) in 41.4% of Caucasians and 1.6% of African-Brazilians, Fy(a−b +) in 37.8% of Caucasians and 17.5% of African-Brazilians and Fy(a−b−) in 1.1% of Caucasians and 66.9% of African-Brazilians [55]. These results indicate that the Manaus/Amazonas region has individuals who express three Duffy phenotypes Fy(a + b +), Fy(a + b−) and Fy(a−b +) more frequently, with expression of FY*A and FY*B antigens.

For the process of parasite invasion into RBCs, Duffy protein is functionally important. In regions of high malaria transmission rates, as in the inhabitants of the Amazon, Duffy protein is naturally immunogenic. Woolley et al. [56] demonstrated (in vitro) that the expression level of the Fy^6^ epitopes was significantly lower in reticulocytes and RBCs from individual carriers of the FY*B/FY*B genotypes compared with individuals of the FY*A/FY*A or FY*A/FY*B genotypes. Another similar study showed that the presence of the FY*BN.01 allele resulted in a 50% reduction of that protein when invaded by *P. vivax* [57].

In a study with *P. vivax* malaria patients, the FY*A and FY*B alleles were found to have low, medium, and high parasitic density [18]. However, in the presence of the GATA variant, genotypes with FY*BN.01 and FY*BW.01 alleles were found only in patients with low parasitic density and low symptomatology.

A study performed in the state of Pará-Brazil, with a population of African descent demonstrated that the presence of the c.202G > A variant was 0.060, the Duffy blood group included 24.3% Duffy negative and 41.3% of the individuals were heterozygous for FY*B^*ES*^ (ES: erythrocyte silent). The frequency of the FY*B^*ES*^ allele was 41.0% [44]. These findings support the monitoring of individuals with G6PD deficiency for use of primaquine during the routine care of afro-descendant communities of the Trombetas, Erepecuru, and Cumná Rivers, in order to assess the risk of haemolytic crisis in recurrent cases of malaria in the region.

Several studies have revealed that the complexity of phenotypic and genotypic variation of the Duffy system and the variants of G6PD (A-) is geographically variable across human populations in areas in which malaria is endemic [26, 58–61]. The Duffy system and G6PD are polymorphic systems that offer great challenges to researchers not only due to their academic importance, but also due to their potential applications to treatment of vivax malaria [62]. Whenever natural selection occurs within a population in an area of endemicity for malaria, natural adaptations may result from genetic variation that provides a partial defense mechanism against *P. vivax* infections [56].

One of the major confounders and a limitation in this study is the inherent disadvantage of G6PD deficiency due to the associated haemolysis, which may be one of the factors that account for the absence of consensus on the G6PD-malaria protection hypothesis. In some cases, this can potentially protect against uncomplicated malaria, but not severe malaria [63, 64]. Despite most G6PD-deficient persons being asymptomatic, haemolytic anaemia, a main clinical sign, can occur 1–3 days after exposure, after eating fava beans, or can be triggered by infections or by certain drugs, such as those used to treat malaria [65, 66]. In many cases, this acute haemolytic anaemia is usually self-limiting, thus, resolving on its own [67]. In addition, the authors acknowledge that an important limitation of this study is the small sample size and they understand that additional studies of larger samples will be required to confirm the results.

## Conclusion

A high prevalence of G6PD c202G > A and c.376A > G and Duffy variants is observed in Manaus, Amazonas state, Brazil, principally in severe vivax malaria patients. This presents a new viewpoint in the protection or not in vivax malaria. In addition, this study reports for the first time the Duffy null phenotype Fy(a−b−) in the population of the Amazonas state. Moreover, it is understood that the relationship between G6PD and Duffy variants can modify clinical symptoms in malaria caused by *P*. *vivax* and this deserves to be further investigated and explored among this population.

## Supplementary Information

**Additional file 1: Table S1**. World Health Organization diagnostic criteria for severe falciparum malaria. Adapted from: Guidelines for the Treatment of Malaria, World Health Organization, 2015.

**Additional file 2: Table S2**. Phenotypic frequency distribution of Duffy blood group among uncomplicated and severe vivax malaria according to gender. No significant correlations were demonstrated in the frequency analysis of the Duffy blood group phenotypes between the gender and uncomplicated and severe P. vivax malaria patients.

## Data Availability

The full data used to support the findings of this study, including, consent terms, electronic files, as well as the lab techniques and materials used may be released upon reasonable request to my Institutional email address: jpmn@ufam.edu.br.
