# Supplementary material for: Duffy blood system and G6PD genetic variants in vivax malaria patients from Manaus, Amazonas, Brazil
Source: Malar J. 2022 May 8;21:144. doi: 10.1186/s12936-022-04165-y (PMC9080172; doi:10.1186/s12936-022-04165-y)
Supplement: Supplementary file 1 — Additional file 1: Table S1. World Health Organization diagnostic criteria for severe falciparum malaria. Adapted from: Guidelines for the Treatment of Malaria, World Health Organization, 2015. [file 12936_2022_4165_MOESM1_ESM.doc]

Supplementary Table 1. World Health Organization diagnostic criteria for severe falciparum malaria.

| **Manifestation** | **Clinical / Laboratory Test** |
| --- | --- |
| Impaired consciousness | A Glasgow coma score < 11 in adults or a Blantyre coma score < 3 in children |
| Prostration | Generalized weakness so that the person is unable to sit, stand or walk without assistance |
| Multiple convulsions | More than two episodes within 24h |
| Acidosis | A base deficit of > 8 mEq/L or, if not available, a plasma bicarbonate level of < 15 mmol/L or venous plasma lactate ≥ 5 mmol/L. Severe acidosis manifests clinically as respiratory distress (rapid, deep, labored breathing). |
| Hypoglycaemia | Blood or plasma glucose < 2.2 mmol/L (<40 mg/dL |
| Severe malarial anaemia | Haemoglobin concentration ≤ 5 g/dL or a haematocrit of ≤ 15% in children < 12 years of age (< 7 g/dL and < 20%, respectively, in adults) with a parasite count > 10 000/μ |
| Renal impairment | Plasma or serum creatinine > 265 μmol/L (3 mg/dL) or blood urea > 20 mmol/ |
| Jaundice | Plasma or serum bilirubin > 50 μmol/L (3 mg/dL) with a parasite count > 100 000/ μ |
| Pulmonary edema | Radiologically confirmed or oxygen saturation < 92% on room air with a respiratory rate > 30/ min, often with chest indrawing and crepitations on auscultation |
| Significant bleeding | Including recurrent or prolonged bleeding from the nose, gums or venipuncture sites; hematemesis or melena |
| Shock | Compensated shock is defined as capillary refill ≥ 3 s or temperature gradient on leg (mid to proximal limb), but no hypotension. Decompensated shock is defined as systolic blood pressure < 70 mm Hg in children or < 80 mmHg in adults, with evidence of impaired perfusion (cool peripheries or prolonged capillary refill) |
| Hyperparasitaemia | P. falciparum parasitaemia > 10% |

Adapted from: Guidelines for the Treatment of Malaria, World Health Organization, 2015 (36,37).
