# Supplementary material for: Duffy blood system and G6PD genetic variants in vivax malaria patients from Manaus, Amazonas, Brazil
Source: Malar J. 2022 May 8;21:144. doi: 10.1186/s12936-022-04165-y (PMC9080172; doi:10.1186/s12936-022-04165-y)
Supplement: Supplementary file 2 — Additional file 2: Table S2. Phenotypic frequency distribution of Duffy blood group among uncomplicated and severe vivax malaria according to gender. No significant correlations were demonstrated in the frequency analysis of the Duffy blood group phenotypes between the gender and uncomplicated and severe P. vivax malaria patients. [file 12936_2022_4165_MOESM2_ESM.doc]

Supplementary Table 2. Phenotypic frequency distribution of Duffy blood group among uncomplicated and severe vivax malaria according to gender.

| Malaria | Male | | | Total | Female | | | Total |
| --- | --- | --- | --- | --- | --- | --- | --- | --- |
|  | Duffy Phenotype  N (%) | | |  | Duffy Phenotype  N (%) | | |  |
|  | Fy(a-b+) | Fy(a+b-) | Fy(a+b+) |  | Fy(a-b+) | Fy(a+b-) | Fy(a+b+) |  |
| Uncomplicated | 13 (27.7%) | 14 (29.8%) | 20 (42.6%) | 47 | 15 (25.4%) | 20 (33.9%) | 24 (40.7%) | 58 |
| Severe | 13 (26.0%) | 19 (38.0%) | 18 (36.0%) | 50 | 15 (22.1%) | 17 (25.0%) | 36 (52.9%) | 68 |
| Total | 26 (26.8%) | 33 (34.0%) | 38 (39.2%) | 97 | 30 (23.6%) | 37 (29.1%) | 60 (47.2%) | 127 |

N: cases
